# QSAR workbench: automating QSAR modeling to drive compound design

**DOI:** 10.1007/s10822-013-9648-4

**Published:** 2013-04-25

**Authors:** Richard Cox, Darren V. S. Green, Christopher N. Luscombe, Noj Malcolm, Stephen D. Pickett

**Affiliations:** 1 Accelrys Ltd., 334 Cambridge Science Park, Cambridge, CB4 0WN UK; 2 Computational and Structural Chemistry, GlaxoSmithKline Medicines Research Centre, Gunnels Wood Road, Stevenage, Hertfordshire, SG1 2NY UK

**Keywords:** QSAR, Workflow, Pipeline pilot

## Abstract

We describe the QSAR Workbench, a system for the building and analysis of QSAR models. The system is built around the Pipeline Pilot workflow tool and provides access to a variety of model building algorithms for both continuous and categorical data. Traditionally models are built on a one by one basis and fully exploring the model space of algorithms and descriptor subsets is a time consuming basis. The QSAR Workbench provides a framework to allow for multiple models to be built over a number of modeling algorithms, descriptor combinations and data splits (training and test sets). Methods to analyze and compare models are provided, enabling the user to select the most appropriate model. The Workbench provides a consistent set of routines for data preparation and chemistry normalization that are also applied for predictions. The Workbench provides a large degree of automation with the ability to publish preconfigured model building workflows for a variety of problem domains, whilst providing experienced users full access to the underlying parameterization if required. Methods are provided to allow for publication of selected models as web services, thus providing integration with the chemistry desktop. We describe the design and implementation of the QSAR Workbench and demonstrate its utility through application to two public domain datasets.

**Electronic supplementary material:**

The online version of this article (doi:10.1007/s10822-013-9648-4) contains supplementary material, which is available to authorized users.

## Introduction

The drug discovery process can be divided into four broad categories: target identification, lead discovery, lead optimization and drug development. The transition from lead optimization to drug development involves the selection of one compound from a series for further evaluation. Thus it is in the lead optimization phase that the important compound properties are defined. Lead optimization is a multi-objective process involving many experimental parameters (assays) relating to target activity, site of action, physico-chemical properties, target selectivity, off-target activities, DMPK and toxicity. Pharmaceutical companies have generated large amounts of data related to many of these end-points and in silico models for QSARs (quantitative structure activity relationships) and QSPRs (Quantitative Structure Property Relationships), can be established to relate the experimental data to computational parameters and chemical (sub)structure descriptors [1, 2]. The models are generated with two end-points in mind: (a) generating an understanding of the properties or chemical features that are correlated with the assay in question to aid in compound design and (b) prediction of multiple assays allowing ranking of compounds prior to synthesis. It should be noted that these two end-points are themselves often in competition—the most predictive model may not be the most interpretable [3–5].

The QSAR model building process can be divided into a number of steps: data preparation, data normalization, descriptor calculation, model building, model validation and model publication. There are guidelines emerging around how to perform each of these steps [6]. Methods for alternative descriptor calculation [7] and model building methods [8] are being sought. However, the over-riding conclusion from these publications is that no descriptor set or model building method will be optimal for all circumstances. This situation is compounded by the fact that many of the modeling methods are available in different software packages with a variety of data formats, front-ends and model export capabilities. This leads to users tending towards the systems they know best or which fit most appropriately with downstream or upstream systems.

Within the context of the pharmaceutical industry, for a model to be useful it must be built in a timely manner, retrospective analysis is of only limited utility to a program, and it must be possible to apply the model (or models) within the standard workflow of the program team. A request from a chemist to a QSAR expert to run a set of predictions on a list of compounds is unlikely to be forthcoming unless the modeler is deeply embedded in the program and can turn around predictions quickly. Better still, the chemist should work with the modeler to understand which models are appropriate and be able to run them as required. The number of available assays and related models coupled with limited expert QSAR resource leads to a continual conflict between the needs of individual programs and the development of more widely applicable global models. There is a need to evaluate any global model in the context of the particular program and to update global models as new data are generated.

Thus new approaches to QSAR modeling are required to address these issues. The DiscoveryBus is one such system developed to allow for a more automated approach to model building through competitive workflow [9]. The AutoQSAR approach can automatically regenerate models as new data become available [10, 11]. AZOrange is an Open Source machine learning platform developed at AstraZeneca [12]. The Automated Modeling Environment (AME) developed at GlaxoSmithKline is another such system [13]. AME provided a slice through the whole modeling workflow, from data gathering from corporate databases through to model publication. However, the system required significant resource to maintain and development and expansion required expert technical skills beyond most QSAR exponents.

In this paper we describe our experiences of QSAR modeling within a pharmaceutical setting and illustrate how these, along with our learning from AME, led us to the development of the QSAR Workbench, a system for automated QSAR data preparation, model building, model validation and model publication. The core of the system is built using a state-of-the-art workflow tool (Pipeline Pilot [14]) and, as such, all important parameters are exposed directly to the QSAR modeler. The system is highly customizable as workflows can be modified or new ones created. Experts can make available well designed model building workflows as starting points for different modeling scenarios with categorical or continuous data and a variety of descriptor sets. This is an important feature of QSAR Workbench as it allows current best practice, as defined by modeling experts, to be captured and then redeployed by less experienced users on their own data sets. The implementation guides the user through the model building process in a straightforward and logical manner. The final published models are themselves workflows and can be published as web services, making them available to end users through the corporate model prediction web-service.

The paper proceeds as follows. In the next section we describe the rationale for our approach, followed by a summary of the implementation of QSAR Workbench with more detail in the supporting information. Next we illustrate the utility of the system with reference to two public domain datasets, toxicity endpoints [15–17] from the CAESAR (Computer Assisted Evaluation of industrial chemical Substances According to Regulations) project [18]. Finally, we conclude with a discussion of our learnings and future steps.

## Rationale

The pharmaceutical industry, GSK included, has a long history in the use of QSAR modeling to support the drug discovery process. The models fall into two broad categories: (1) global models that are built on diverse datasets of potentially tens of thousands of data points and are of general applicability, (2) local models built specifically for a series or project. Local models may be built on the same end points as the global models, where for example the global model is less performant or there is a significant shift in either the gradient (for a linear model) or the magnitude of prediction. For example, it is not uncommon to find series of compounds where the global model preserves the trend but the prediction is shifted. Local models are also relevant to target and selectivity type modeling.

**Fig. 1:**
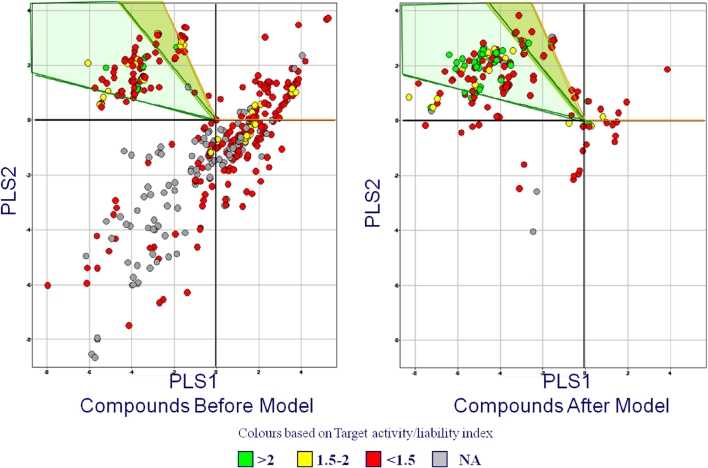
Example application of QSAR modeling to direct a program team to appropriate regions of chemical space. A PLS model was built on models for target activity and liability end-points (P450s, hERG). The *shaded area* was identified as the most relevant and the chemistry team was able to focus its efforts on synthesizing compounds in this region

The individual models may themselves be combined into other models to guide compound design. An example is shown in Fig. 1, which illustrates the use of a model of models to help focus a program into the appropriate regions of chemical space. It is in such applications that QSAR experts add real value to the program teams and hence there is a need to make the building of individual models as straightforward as possible without sacrificing quality. This allows the modeler to focus on the important tasks of assessing and critiquing models and applying them to real world problems.

**Fig. 2:**
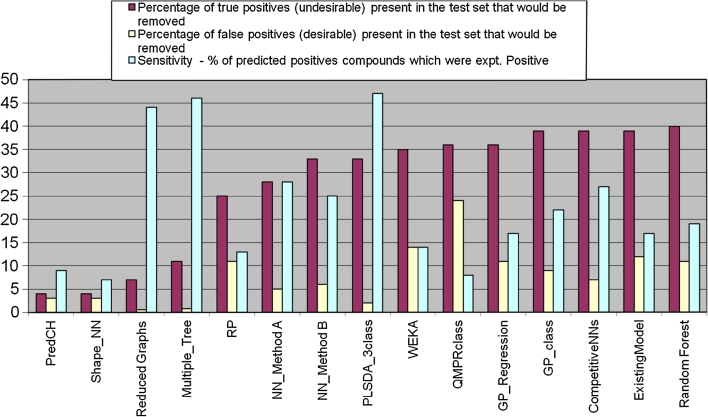
Many person months of effort were required to produce a diverse set of models for Cytochrome P450 3A4 inhibition, covering different descriptor and modeling methodologies

The modeler is faced with a large number of decisions in relation to model building, choice of descriptors and modeling methods being just two. Figure 2 illustrates the scale of the problem. Several years ago we undertook an exercise to evaluate the performance of various modeling methods and descriptors for modeling of Cytochrome P450 3A4 inhibition. The models showed a range of performance in terms of specificity and sensitivity, the choice of which would depend on the application domain. The PLSDA_3class model stands out as having a reasonable balance of specificity and sensitivity, though other models could be more appropriate in specific applications. Thus the ability to generate a range of models with multiple modeling methodologies would be advantageous. However, it took many FTE months of work to generate and analyze these models.

Thus, whilst both these examples are great science they do not scale. A third issue relates to an earlier point, for a model to be useful it needs to be both timely and applicable. At GSK we have a SOAP web-service system that allows us to deploy models that chemists can access through web-based tools and in applications such as Helium [19]. There are currently over 50 global models and a similar number of local models available to aid in compound design at GSK. Maintaining, validating and updating these models present significant issues and could easily take the resource of several highly skilled FTEs. It is these three factors that have led us to look at mechanisms for bringing a greater degree of standardization and automation to the QSAR modeling process.

An interesting perspective on QSAR can be gained by casting the problem in the light of the CRISP-DM paradigm [20]. We have used this approach previously when considering HTS data mining [21]. Within the CRISP-DM model the process can be broken down into six steps: (1) business understanding, (2) data understanding, (3) data preparation, (4) modeling, (5) evaluation, (6) deployment. Clearly steps (1) and (2) rely on the modeler being closely integrated with the program team and having a good understanding of which models are required and how they are being applied. It is our belief that within a mature field such as QSAR modeling it should be possible to design systems that can make steps 3, 4 and 6 as straightforward as possible and provide all the necessary tools and statistics to enable 5. Furthermore, we would suggest that such a system not only enables good science but can actually promote better science as the expert is freed up to focus on the key aspects of the problem and applying models in real world situations.

AME [13] represented our first approach to building such a system. This was a fully functional system that took data from the corporate repository, built models and had the ability to publish models to the internal web-service. Using this system we were able to build 11,000 models across 326 endpoints in a period of weeks. The utility goes far beyond the individual models, allowing many strategic questions to be addressed: are there subsets of descriptors that work best, do some statistical methods work better than others, are there combinations of descriptors and methods, do larger data sets lead to better models and so on? The main problem with AME was one of supportability. It required full time IT support to maintain and implement new methods with the result that the modeler had the impression of using a black box.

QSAR Workbench represents our progression to the next generation of automated QSAR model building that addresses many of the issues described above. In the next section we describe the system in more detail, with a fuller technical implementation in the supporting information.

## QSAR Workbench implementation

### Overview

A fuller description of the system, with example screenshots, is provided in the supporting information. Here we shall outline the main design objectives and features of the system. The subsequent example applications provide some more detail on points of particular relevance.

Our goals in designing the QSAR Workbench were: (a) to decouple the scientific workflows from the User Interface so that the QSAR expert could maintain control over the important features and avoid the black-box; (b) to provide a rich environment for evaluating and triaging models; (c) to build individual models rather than employ competitive modeling workflows or in any other way automatically select models, the user sees all models and uses their own judgment, guided by the plots and statistics, on the most appropriate model for the intended use; (d) to have the ability to publish models for general use; (e) to publish validated model building workflows, or subsets thereof, so that users can quickly assess the model landscape and gain insight into what sorts of models and descriptor sets are likely to be most applicable (if any) to the problem at hand; (f) to capture the output from the modeling process in a manner that permits easy communication of how the model is built, model performance and potentially important descriptors.

Pipeline Pilot seemed an ideal candidate as an environment in which to build the application because of existing web-service integration, the availability of a number of QSAR modeling methods, links to third-party software such as R and a set of rich reporting tools.

Calculations presented in this paper were performed on a single node of a 64-bit Quad-core Windows laptop with Intel i7 processors running at 1.73 GHz, with 16 GB RAM.

### Application design

QSAR Workbench is a lightweight Pipeline Pilot web application that provides an intuitive, user centric, sandbox environment for building, validating and publishing QSAR models. Although aimed at workgroup sized teams of users, the application also provides enterprise scale capabilities such as integration points via Web Services for existing corporate modeling applications and workflow capture and replay.

QSAR Workbench is a JavaScript based Rich Internet Application (RIA) [22, 23] where the majority of the application’s code resides in the client tier whilst the server side layer of the application is simply responsible for providing data (usually formatted as XML, [24] JSON [25] or HTML [26]) to the client layer, this application design is commonly referred to as AJAX [27]. QSAR Workbench makes extensive use of the Pipeline Pilot Enterprise Server as an application server; for example to provide JSON formatted data to the client application, as a scientific modeling platform; to provide services to build and validate models using several learner algorithms and also as a reporting server to return HTML formatted data to the client. The implementation uses the Pipeline Pilot Client SDK (Software Development Kit) which allows communication between the client and Pipeline Pilot via SOAP [28] Web Services [29] and also several extensions to the SDK to provide tight integration with a third party JavaScript library. The workbench utilizes a custom extension to the Pipeline Pilot reporting collection which allows for flexible client side validation of HTML forms. The web application is decoupled from the underlying modeling workflows. The web forms and computational processes are created and run by standard Pipeline Pilot protocols making it straightforward for anyone familiar with Pipeline Pilot to update and maintain the core science protocols e.g. to add new descriptors or modeling methods. The web application locates these protocols from specific folders on the server, making it possible to add new processes without modifying the user interface directly.

QSAR Workbench is organized as a user-based system, with each user managing a set of projects. On loading a dataset of structures and endpoint to be modeled the user has two options. The user can run one of a number of preconfigured validated protocols that can encapsulate part or all of the modeling process as described below. Thus with a single button click the system can build models and present the results, giving the user feedback on the system’s ability to model the endpoint. Alternatively the user can follow a guided workflow that takes them through each stage of the process in a more manual and customizable fashion. The modeling workflow consists of the following steps: (1) Prepare data, (2) Split data, (3) Descriptors, (4) Build Model, (5) Validate Model, (6) Publish. We describe each of these briefly.

#### Prepare data

This allows the user to apply appropriate desalting, chemistry normalization and standardization to business rules compatible with the descriptor definitions. The response property can be normalized in a number of ways including scaling to unit variance or log transformation. Continuous data can be converted to categorical and binary categories created.

#### Split data

Several algorithms are provided for splitting the data into test and validation sets: clustering on chemical fingerprints or properties, stratification according to activity or random splits can be generated. Several different methods can be applied within the scope of the project and models will be built over all of them. Tools are also provided to visualize the splits in a user-defined property space and these also allow the user to generate a manual split if desired.

#### Descriptors

A full range of standard 1D, 2D and 3D descriptors are available as listed in the supporting information. These include physicochemical property calculators, e-State values, topological indices as provided by standard Pipeline Pilot components. The system has a user extension allowing the inclusion of additional descriptors and it is straightforward to replace the standard descriptors, e.g. for calculated logP, with a preferred version. Once descriptors are calculated, sets of descriptor subsets can be created and models can be built over each subset.

#### Build model

The system provides access to a set of modeling techniques as shown in Table 1.

**Table 1:** Statistical learners available in QSAR Workbench

Statistical method	Categorical	Continuous	Details
PP Bayes	Yes	No	Naïve Bayes as implemented in Pipeline Pilot [41]
PP RP forest	Yes	No	Recursive partitioning forest model [42] as implemented in Pipeline Pilot
PP RP tree	Yes	No	Recursive partitioning tree model [42] as implemented in Pipeline Pilot
R NN	Yes	Yes	Neural network model as implemented in R package nnet [43]
R SVM	Yes	Yes	Support vector machine model as implemented in R package e1071 [44]
PP PLS	No	Yes	Partial least squares model as implemented in Pipeline Pilot
R PLS	No	Yes	Partial least squares model as implemented in R package pls [45]

These provide access to a range of linear and non-linear methods. The user can select one or more methods and models are built over the combination of data splits, descriptors and modeling methods to provide a matrix of models. Note that we have taken the decision not to employ competitive workflows in building these models. The choice of the appropriate model is left to the user. The model’s own internal cross-validation or train/test split (if any) is applied to the QSAR Workbench Training Split when building the model.

#### Validate model

This is the most critical part of the process, providing tools for validating models and evaluating a potentially large model landscape. The models are applied to the QSAR Workbench Test Split and a number of statistics and plots are automatically generated to allow the user to compare the models. The results view contains summary plots appropriate to the modeling endpoint (categorical or continuous). These include a ROC [30] or REC [31] curve and other plots allowing a quick visualization and assessment of model performance.

The intention is to provide the user with an overview of the overall performance of the various model combinations, as well as highlighting potential informative features such as a particular learner that always models well, or one model that performs particularly better than the others, indicating potential over-fitting or other issues. The issue of finding chance correlations when running such a process should not be overlooked and these summary plots provide some reassurance that you do not end up focusing on one outlier model that happens to have good statistics by chance. Below the summary plots is a spreadsheet view containing more detailed statistics. This is sortable and allows the user to select models for more detailed analysis. The detailed view provides a mechanism for comparing a small number of models and making a choice as to which, if any, to publish. Validate model also provides methods for running predictions against additional external validation sets such as temporal datasets.

#### Publish

If a model of sufficient quality is obtained, the user can choose to publish it. This makes the model available to all users in Pipeline Pilot. More importantly the publishing process provides access to the model by external web-services. This uses the fact that Pipeline Pilot protocols are themselves web-services and protocols are provided to list published models and apply them to user-supplied datasets.

One of the key features of the QSAR Workbench is the ability to remember and document the steps taken in building the model. These steps are applied when publishing the model so that the same data preparation steps are automatically applied when making predictions. In addition, a model report is automatically generated as a PDF document and also included in the model component help text. The model report contains full details on the model building process and performance, thus providing appropriate documentation for inclusion in electronic Lab Notebooks and to link from prediction tools to allow users to see the model details.

In addition to models, users can publish the model building protocol, consisting of one or more of the sub-steps involved in creating a model (or models). Thus the user can publish a protocol that automates just the chemistry preparation stage or can build all available models against a categorical endpoint. These are available to other users and can be accessed via the front page on loading a dataset.

## Use cases

As an example of the use of the QSAR Workbench we have chosen to revisit two of the five environmental toxicology endpoints previously modeled as part of the CAESAR initiative [15–18]. We will show how use of the QSAR Workbench framework can enable practical exploration of a large model space, identifying potential outlier models, and trends and biases caused by specific descriptors, statistical methods or, more commonly, training/test set splits. The rich reporting available in the model triage allows identification of the pros and cons of individual models beyond standard statistical metrics.

### Exploring model space

For the purpose of consistency we have chosen to explore identical descriptor and training/test set splits for all models. Each dataset was split into a training set and a test set at two different percentages for three of the available algorithms described in the supplementary material. The algorithms chosen were Random, Individual Clusters (Optimized) and Random Per Cluster. This results in six different splits for each endpoint. In addition we have utilized the training/test set split as defined in the QSAR Model Report Format (QMRF) submissions for the two endpoints studied, available from the CAESAR web-site [32]. Table 2 provides a summary of all splits used, and the labels used to reference them in subsequent discussion.

**Table 2:** Details of training/test set splits used for all endpoints. For details of split algorithms see the data set splitting section above and in the supplementary material

Split label	Training set percentage	Split algorithm
Rand50	50	Random
Rand75	75	Random
IndOpt50	50	Independent clusters (optimized)
IndOpt75	75	Independent clusters (optimized)
RPC50	50	Random per cluster
RPC75	75	Random per cluster
From Paper	80	As defined in the QMRF submissions

To explore descriptor space we have selected a total of 194 2-dimensional descriptors. Full details of the descriptors are provided as supporting information. The total descriptor set was partitioned into ten descriptor subsets. Table 3 gives a summary of the descriptor subsets, and the labels used to reference them in subsequent discussion.

**Table 3:** Details of descriptor subsets used for all endpoints. Further details on the descriptors are given in supporting information

Descriptor subset	Number of descriptors	Descriptor subset details
Chi	12	Kier-Hall topological Chi indices [46]
ECFP6	1	Extended connectivity fingerprint with atom type classes, diameter 6 [47]
Estate	161	Electrotopological state values and counts [48–50]
FCFP4	1	Extended connectivity fingerprint with functional type classes, diameter 4 [47]
Molprops	19	A set of simple common molecular properties and counts as implemented in Pipeline Pilot
Chi_Molprops	31	Combination of subsets Chi and MolProps
ECFP6_Molprops	20	Combination of subsets ECFP6 and MolProps
Estate_Molprops	180	Combination of subsets estate and MolProps
FCFP4_Molprops	20	Combination of subsets FCFP4 and MolProps
Chi_ECFP6_Estate_FCFP4_Molprops	194	Combination of subsets Chi, ECFP6, estate, FCFP4 and MolProps

We have employed all relevant statistical model learner methods for each endpoint as defined in Table 1. For categorical endpoints there are five methods, for continuous endpoints four methods.

For each endpoint we have explored the full combinatorial model space available from the combination of splits, descriptor subsets and learner methods detailed above. For categorical endpoints this gives rise to a potential model space of 350 models, for continuous endpoints 280 models. The QSAR Workbench also allows a fourth dimension of model space to be explored, namely the underlying parameters of the learner methods. However for the sake of this work we have excluded this dimension, and simply exploited the default parameters for the underlying Pipeline Pilot implementation of each method.

### Skin sensitization classification models

**Fig. 3:**
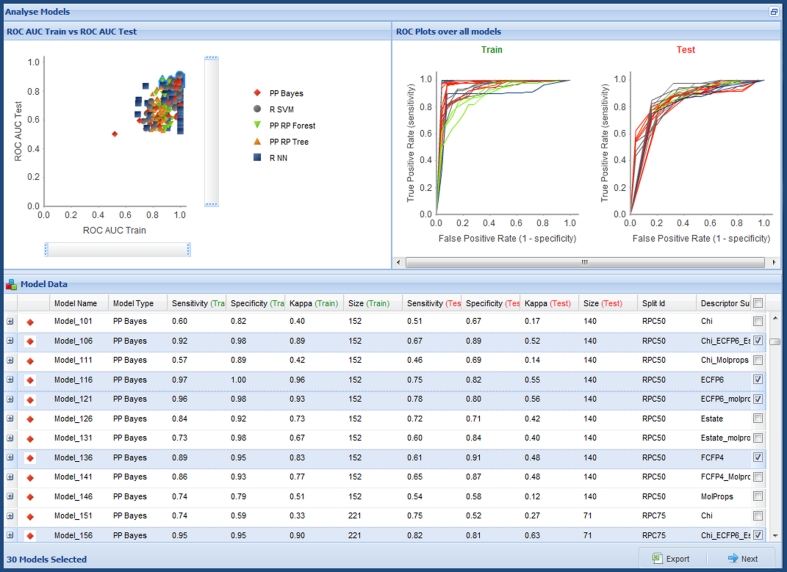
Model validation and triage view. The view shown is for categorical models. The summary plot of AUC test versus train is scaleable. The right-hand plots update with the selected models in the spreadsheet. Models are grouped by model type and are sortable by the different columns. Selected models can be progressed to a more detailed view, providing more information on individual models

Assessment of skin sensitization potential is a requirement under REACH Annex VII. Structural data were provided by the original authors. The raw data are classified into five categories: non; weak; moderate, strong; extreme. Though the majority of statistical methods currently exposed through the QSAR Workbench are able to build multi class models, we have chosen to follow the work of Chaudry et al. [15] who built binary classifiers. All results presented in this work have been defined using a “non-sensitizer” class containing all compounds with raw classification “non” or “weak”, resulting in a total of 108 non-sensitizers and 101 sensitizers. For the purposes of statistical calculation—e.g. model specificity—the positive class is defined as “sensitizer” (Fig. 3)

**Fig. 4:**
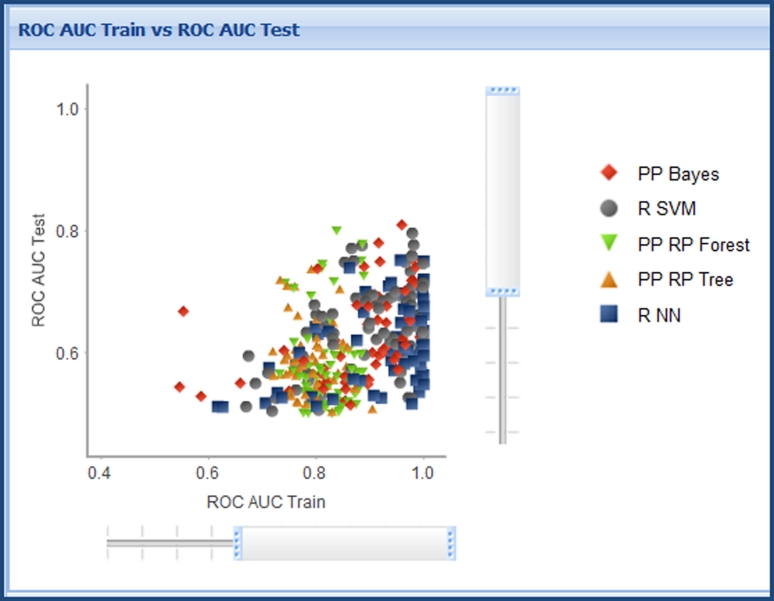
ROC plot for training versus test set for all models built for the skin sensitization end point

**Fig. 5:**
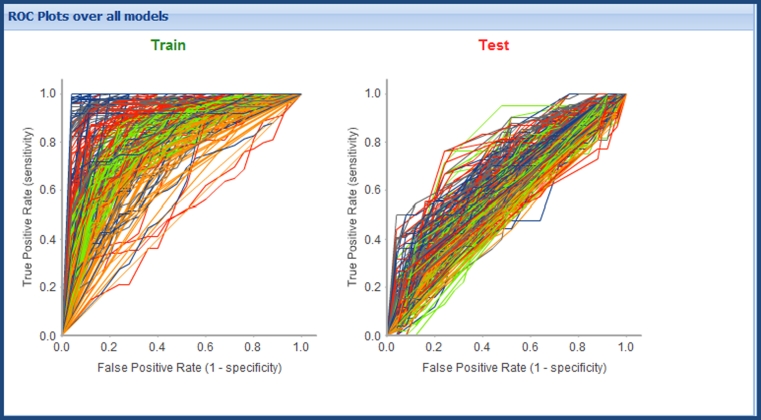
Comparative ROC plots for training and test set for all models built for the skin sensitization endpoint. The color scheme for the models is the same as that in Fig. 4

Of the potential 350 models, 346 were successfully built. Plots of Train versus Test ROC AUC and comparative ROC plots over the successfully built models are shown in Figs. 4 and 5 respectively. All models are shown for illustration, however, the interactive triage in the Workbench means that we would rarely look at the full set of results in this way, more commonly filtering down to a few models of interest. From Fig. 4 it is possible to observe a potential trend in terms of models exhibiting over-fitting. There is a clear set of models with very high training set ROC AUC (~1.0) yet a broad range of values for test set ROC AUC, these are built mostly using the R NN learner method. The interactive nature of the model triage report allows selection of a subset of models from the chart. Selection of the models with a ROC AUC score of ~1.0 for the training set and passing these through to the second step of the model triage report reinforces the impression given by the comparative chart: of the 39 selected models, 28 were built using the R NN method; 3 with PP Bayes; 8 with R SVM. Analysis of the different train/test set splits used in these likely over-fit models shows no overall trend, 4 models use From Paper; 10 IndOpt50; 4 IndOpt75; 9 Rand50; 3 Rand75; 4 RPC50; 5 RPC75. Again there is no obvious trend in the descriptor subsets used, 8 models use Chi_ECFP6_Estate_FCFP4_MolProps; 7 ECFP6; 4 Estate_molprops; 5 FCFP4; 8 FCFP4_Molprops.

This analysis might lead us to distrust the models built using the R NN method in the context of this study. However it is most likely that the reason for this arises from a sub-optimal choice of learner parameters for modeling this data. In real-life application of the QSAR Workbench the modeler would almost certainly re-visit this dimension of model space to understand better the root cause. Here we shall simply discount these—and other R NN derived models—from further consideration.

Returning to the first step of the model triage report, we now investigate the small set of “best” models—those with high ROC AUC for both training and test sets. Again we look at the break down of statistical method, training/test set splits and descriptor subsets. Of the 5 models, 2 are built using PP Bayes and 3 with R SVM. Considering the training/test set splits: four use From Paper and one RPC 75. In the descriptor subset dimension the model breakdown is as follows: three use Chi_ECFP6_Estate_FCFP4_Molprops; one ECFP6_Molprops; one FCFP4_Molprops. Though this subset of models is almost certainly too small to draw any concrete conclusions, it already raises some suspicions. The training/test set split From Paper was generated in a very similar manner to RPC75—“by random but stratified, sampling” [15]. Though this mechanism for defining a test set is commonly employed, there is a risk of over-emphasizing the model quality from test set statistics. Because the test set is selected as a subset of structurally derived clusters, there is an explicit similarity between the training set and test set. This lack of independence means one would generally expect the test set statistics to be of similar quality to the training set.

**Fig. 6:**
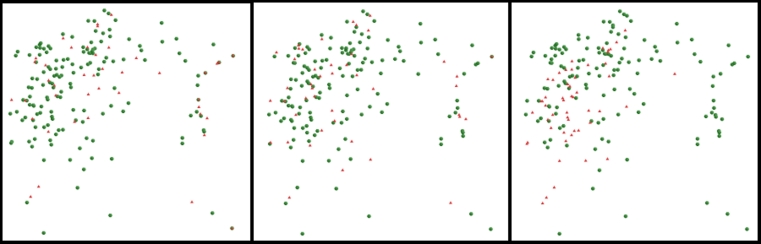
Comparison of data set splits in chemical space for the skin sensitization end-point, using multi-dimensional scaling plots based on ECFP_6 fingerprint similarity for: From Paper (*left panel*); RPC75 (*centre panel*); IndOpt75 (*right panel*). Training set members are represented by *green circles*, test set members by *red triangles*

To examine this effect the QSAR Workbench provides analysis tools for visualizing the training/test set splits in a two-dimensional representation of structural similarity (the Analyze Split task). In Fig. 6 three such plots are shown, these have been generated using the ECFP_6 fingerprint. It is immediately obvious that the From Paper and RPC75 splits have very similar distributions, with test set compounds evenly interspersed with training set compounds. In contrast the IndOpt75 plot shows clear “islands” containing just training or just test set compounds. So, although these five “best” models seem to show robust statistics for both training and test sets, an experienced modeler looking to build a model with reliable predictive power, may not be entirely happy with any of them. Thus, we now look at models with balanced training/test-set statistics in the hope that the final model would provide greater predictive power in real-life application.

**Fig. 7:**
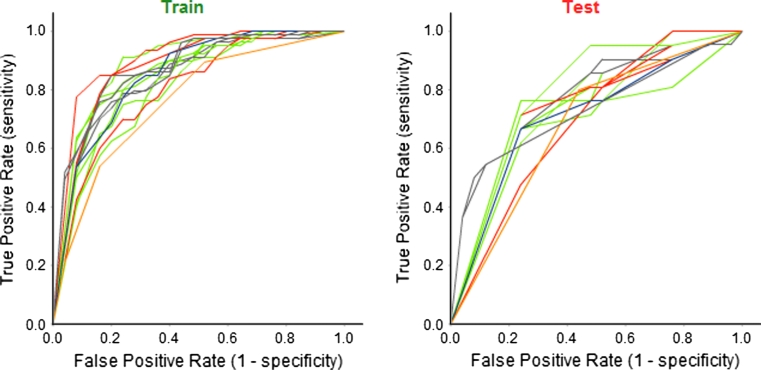
Filtered set of comparative ROC plots for “balanced” models for the skin sensitization end-point

A “balanced” set of models was selected from step one of the model triage report. Figure 7 shows the filtered set of comparative ROC plots for training and test sets. This subset of 16 models shows no overall trend in terms of statistical method, containing three models built using PP Bayes; 6 with PP RP Forest; 2 with PP RP Tree; 1 with R NN; 4 with R SVM. As with the “best” model set, these models do show a strong preference for training/test set splits defined with random selection within individual clusters: 12 models use From Paper; two IndOpt75; two RPC75. The descriptor subsets used show no major overall trend: 3 use Chi_ECFP6_Estate_FCFP4_Molprops; 1 ECFP6_Molprops; 3 Estate; 4 Estate_Molprops; 1 FCFP4; 2 FCFP4_Molprops; 4 Molprops. However it is interesting to note that 12 of the 16 models include the simple set of molecular properties and property counts—the subset Molprops. This is an appealing result as these descriptors are simple to interpret—an important factor in judging the quality of QSAR models according to the OECD principles [33]. Following the discussion about the “best” model set, we here chose to set aside models built using the From Paper and RPC75 splits.

**Fig. 8:**
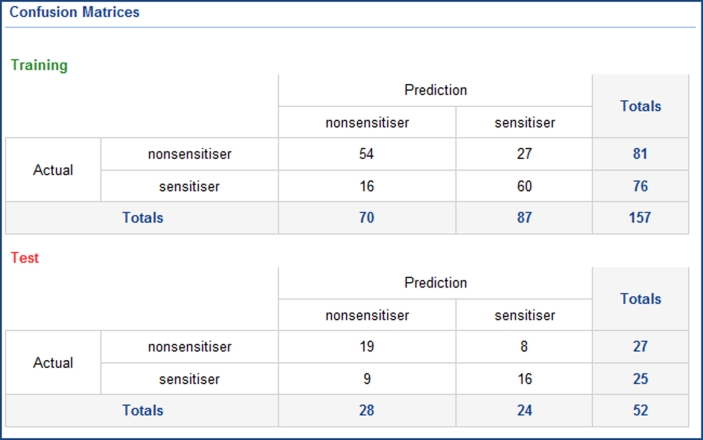
Confusion matrix for the skin sensitization model built using the PP RP Tree learner method, IndOpt75 training/test set split and MolProps descriptor subset

The two remaining models, which both use the IndOpt75 split, and the same learner method (PP RP Tree), have identical statistical measures, and confusion matrices. As such the preference would be for the model using the simplest descriptor subset, MolProps. The confusion matrix for this model is shown in Fig. 8. An appealing aspect of the use of the PP RP Tree method is that we can get a direct understanding of the importance of individual descriptors to the final model. Table 4 shows the “Number of Questions” in which each of the 19 descriptors within the MolProps subset is used, each question representing a branch within the tree. As can be seen this model could be further simplified with little loss of quality by only considering the five most significant descriptors: ALogP; Molecular_Weight; Num_RotatableBonds; Num_Bonds; Num_Atoms. Table 5 shows comparative statistics for several models: this manually selected “balanced” model—using PP RP Tree/IndOpt75/MolProps; the model with the best test-set ROC AUC—using PP Bayes/FromPaper/Chi_ECFP6_Estate_FCFP4_MolProps; the published model of Chaudry et al. [15].

**Table 4:** Frequency of descriptor usage in the skin sensitization model built using PP RP tree method, IndOpt75 split and the Molprops descriptor subset

Descriptor	Number of questions
ALogP	17
Molecular_Weight	17
Num_Rotatablebonds	16
Num_Bonds	13
Num_Atoms	12
Num_H_Acceptors	4
Num_H_Acceptors_Lipinski	4
HBA_Count	3
Num_H_Donors_Lipinski	3
HBD_Count	2
Num_H_Donors	2
Num_Rings	2
Num_StereoBonds	2
Num_AromaticBonds	1
Num_AromaticRings	1
Num_StereoAtoms	1
Num_BridgeBonds	0
Num_BridgeHeadAtoms	0
Num_SpiroAtoms	0

**Table 5:** Comparison of model statistics for selected models for the skin sensitization end-point. See main text for details of model selection

Model details learner/split/descriptor	Training set sensitivity	Training set specificity	Test set sensitivity	Test set specificity
PP RP Tree/IndOpt75/MolProps	0.79	0.67	0.64	0.70
PP Bayes/From Paper/Chi_ECFP6_Estate_FCFP4_MolProps	0.86	0.93	0.67	0.90
Chaudry et al. [15]	0.85	0.87	0.70	0.67

### Bioconcentration factor regression models

Bioconcentration factor (BCF) describes the likelihood of chemical concentration in organisms due to environmental exposure to the compound. Assessment of BCF is a requirement within REACH legislation. We have taken structural data from the curated data set of Lombardo et al. [16, 17], published as part of their QMRF submission.

**Fig. 9:**
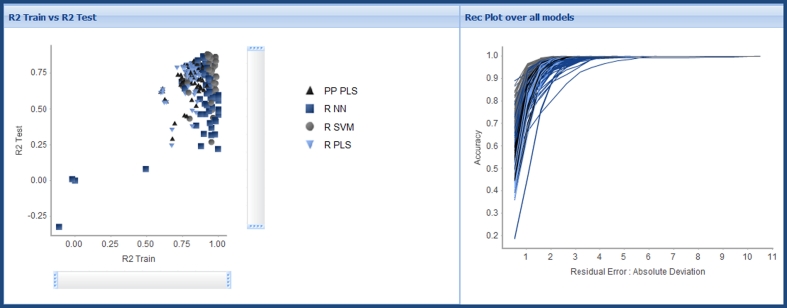
Training set versus test set R^2^ (*left*) and REC plot over all models built for the BCF endpoint

Data preparation, train/test splits and model parameters were as described above for the Skin Sensitization models. Of the potential 280 models, all were successfully built. Plots of Train versus Test R^2^ and comparative REC plots over the successfully built models, as presented to users in step one of the model triage report, are shown in Fig. 9. The purpose of these plots is to give the modeler an overview of relative model performance and to indicate modeling methods, splits and/or descriptors that are outliers or may be overfit. There are issues with using R^2^ (or any statistical measure) in isolation to select between models, particularly with varying data set splits and sizes. This is mitigated for by the REC plot and the inclusion of other statistical parameters such as RMSE in the sortable table below the plot (as illustrated in Fig. 3). More detailed analysis is provided in subsequent visualizations following model selection.

In contrast to the classification models built for the skin sensitization end-point there are no clear trends, in terms of over-fitting, observable from these plots. It does appear however that there are a large number of high quality models that use the R SVM learner. Selection of the best models and drill-down through step two of the model triage report confirms this observation. Of the 51 selected models 30 are built using the R SVM learner; 5 with PP PLS; 14 with R NN; 1 with R PLS. Comparison of the distribution of the different training/test set splits methods shows no clear trend, with 6 of the 7 methods contributing to this subset of models: 8 models use Chi_ECFP6_Estate_FCFP4_MolProps; 9 Chi_MolProps; 3 ECFP6_MolProps; 6 Estate; 11 Estate_MolProps; 2 FCFP4_MolProps; 11 MolProps. It is interesting to note that 88 % of these models are built with descriptor sub-sets containing the MolProps set of simple molecular properties.

**Fig. 10:**
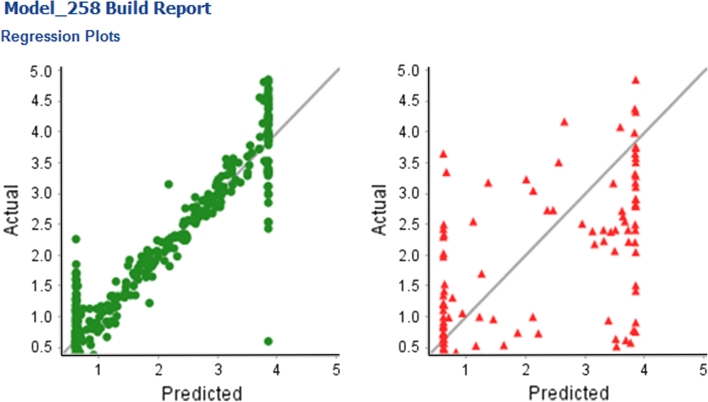
Actual versus predicted regression plots for Model_258, for the BCF end-point. Results for the training set are shown on the *left*, results for the test set on the *right*. This is an example of a poor model even though the model statistics appear reasonable

Using the drill-down to fine details of the models available from step two of the model triage report, allows identification of poor models which might have been considered reasonable if simply considering the raw statistical metrics. An example of such a model is Model_258, this model was built using R NN learner, Rand75 split method and ECFCP6_MolProps descriptor subset. The training set R^2^ of 0.94 and RMSE of 0.46 is very promising (the variability in experimental values is 0.45 log units), though the test set values of 0.62 and 1.24 for R^2^ and RMSE are less encouraging. The plots of predicted versus actual response, shown in Fig. 10, highlight some serious issues with this model. For both training and test sets the model appears to have strict upper and lower boundaries on predictions.

We now analyze the distribution of the different learner algorithms, training test set splits and descriptor subsets, for three hierarchical subsets of models, with increasing test set R^2^. 175 models have a test set R^2^ of 0.70 or better, but there appears to be no overall trend. Of these models 43 are built using PP PLS; 30 with R NN; 49 with R PLS; 53 with R SVM. Considering the different training/test set splits: 28 use From Paper; 15 IndOpt50; 16 IndOpt75; 29 Rand75; 29 Rand75; 29 RPC50; 29 RPC75. The distribution of descriptor subsets used is: 5 use the Chi subset; 18 Chi_ECFP6_Estate_FCFP4_MolProps; 25 Chi_MolProps; 2 ECFP6; 20 ECFP6_MolProps; 22 Estate; 25 Estate_MolProps; 12 FCFP4; 20 FCFP4_MolProps; 16 MolProps. We next consider the subset of these models that have a test set R^2^ of 0.80 or better, 79 models. Of these models 16 are built using PP PLS; 16 with R NN; 11 with R PLS; 36 with R SVM. Considering the different training/test set splits: 12 use From Paper; 2 IndOpt50; 9 Rand50; 12 Rand75; 21 RPC50; 23 RPC75. The distribution of descriptor subsets used is: 9 use the Chi_ECFP6_Estate_FCFP4_MolProps split; 13 Chi_MolProps; 10 ECFP6_MolProps; 12 Estate; 13 Estate_MolProps; 9 FCFP4_MolProps; 13 MolProps. Finally reducing this subset to only consider models with test set R^2^ of 0.85 or better leaves us with just 25 models. Of these models one is built using PP PLS; 4 with R NN; 20 with R SVM. Considering the different training/test set splits: 4 use FromPaper; 5 Rand50; 4 Rand75; 4 RPC50; 7 RPC75. The distribution of descriptor subsets used is: 4 use the Chi_ECFP6_Estate_FCFP4_MolProps split; 5 Chi_MolProps; 4 Estate; 4 Estate_MolProps; 8 MolProps.

**Fig. 11:**
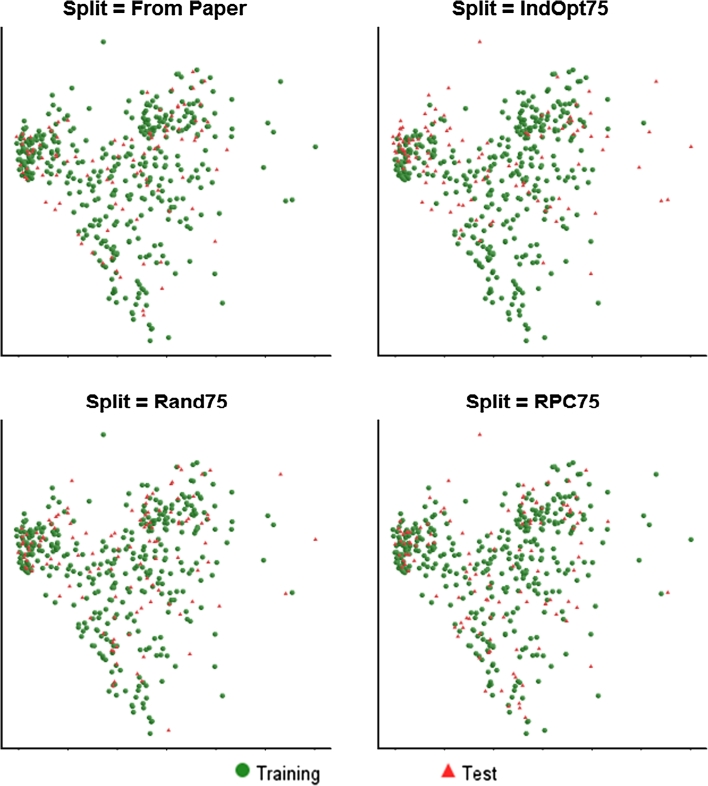
Comparison of data set splits in chemical space for the BCF end-point, using multi-dimensional scaling plots based on ECFP_6 fingerprint similarity for: From Paper (*top*-*left*); IndOpt75 (*top*-*right*); Rand75 (*bottom*-*left*); RPC75 (*bottom*-*right*). Training set members are represented by *green circles*, test set members by *red triangles*

As the set of models retained is reduced across the three cut-offs for test set R^2^ we can begin to identify some trends. Firstly in terms of the “best” learner method, the R SVM method clearly stands out, being used in 30, 46 and 80 % of the models for the 0.70, 0.80 and 0.85 cut-offs respectively. The frequency that we see use of the different splits, follows what is probably an unsurprising trend, with the number of models using either of the IndOpt methods falling from 18 % at the 0.70 cut-off to 2.5 % at the 0.80 cut-off, and no models using this method at the 0.85 cut-off. As discussed in the analysis of the results for the skin sensitization model, the IndOpt split selection method explicitly attempts to make the test set “look” different (in terms of chemistry space) to the training set. The split methods that ensure that the test set do look similar to the training set tend to out-perform the IndOpt method, when considering test set statistics. These methods include the RPC splits, and the From Paper split—which in this case is an 80:20 random selection [16]. The QSAR Workbench Analyze Splits tasks can again be used to compare to known methods. Figure 11 shows comparison of four split methods for the BCF data set. Though not as pronounced as in the skin sensitization example, the plot for the IndOpt75 split does show islands of compounds in the test set separated from any compounds in the training set, for example the five compounds on the far right of the plot, and a smaller island on the top-left of the plot. There are again marked similarities between the plots for the From Paper and RPC75 splits. There does not appear to be any major trend in the distribution of descriptor sub-sets used across the increasingly more accurate model sub-sets, though it is again pleasing to note that all but 4 of the most accurate 25 models include, at least in part, the simple MolProps descriptor subset.

**Fig. 12:**
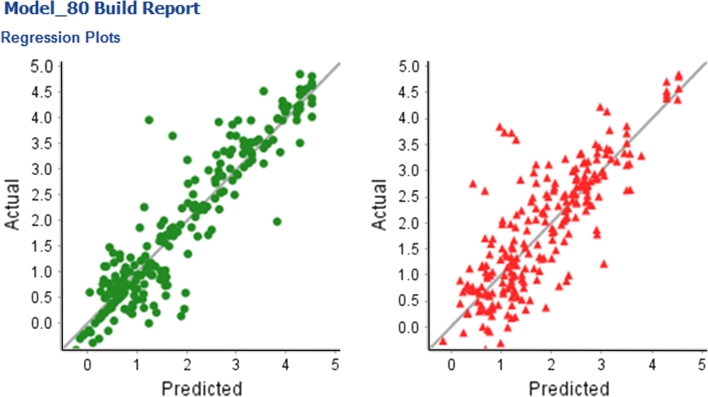
Actual versus predicted regression plots for Model_80, for the BCF end-point. Results for the training set are shown on the *left*, results for the test set on the *right*. Model_80 was built using R SVM, IndOpt50 split and MolProps descriptor subset

Table 6 shows the training and test set R^2^ and RMSE for four models: the model published by Lombardo et al.; the best model (in terms of test set R^2^) found using the From Paper split; a “balanced” model, which uses the IndOpt split method, thus eliminating explicit bias towards improved test set statistics; the best model (in terms of R^2^) found excluding those built with either the From Paper or RPC split methods, in this case using the Rand75 split method, where it is not entirely clear what bias may have been introduced. It should be noted that the model of Lombardo et al. is actually a composite model composed of two different predictors. In addition a domain of applicability test was employed so that not all compounds in the test set were included in the statistics of the published model. All of the models created for this work use the R SVM learner, two of the three use just the MolProps descriptor sub-set, the other the Chi_MolProps subset. All of these models show very reasonable statistics, as such it would probably be appealing to use the one without bias in the test-set statistics for real-life application. Figure 12 shows the actual versus predicted regression plots for training and test sets for this finally selected model.

**Table 6:** Comparison of model statistics for selected models for the BCF end-point. See main text for details of model selection

Model details	Training set R^2^	Training set RMSE	Test set R^2^	Test set RMSE
Lombardo et al.	0.85	0.53	0.83	0.51
R SVM/Rand75/Chi_MolProps	0.93	0.49	0.88	0.65
R SVM/FromPaper/MolProps	0.93	0.50	0.86	0.68
R SVM/IndOpt50/MolProps	0.93	0.52	0.81	0.70

## Discussion and conclusions

The QSAR Workbench encapsulates the workflow required to build, validate, analyze and publish QSAR models. The intended users are QSAR model experts, where the system provides a framework to build and explore a range of configuration and model building parameters, and modelers with an understanding of the principles of QSAR but perhaps with less familiarity of the various algorithms and software packages available.

The system can be used in a highly automated fashion through the configuration of appropriate default settings for different modeling scenarios: continuous, categorical, multi-class data sets. These different scenarios would normally require expert parameterization and set up using a variety of bespoke and/or 3rd party software but through the Workbench the appropriate best practice can now be embedded into saved workflows for immediate selection and replay on new data sets of similar nature by users with only limited knowledge of QSAR. Thus even the relatively inexperienced modeler will be capable of exploring good quality QSAR solutions to their data with the confidence that the modeling has been appropriately set up. In this scenario the system can provide a rapid assessment of whether a particular dataset is amenable to QSAR modeling and point the user to model subspaces worthy of further exploration. In the use cases presented in this work, the human set-up time to build the approximately 600 QSAR models was under one hour, once the model spaces have been built the modeler is able to concentrate on the analysis of the models.

The publication capabilities of the QSAR Workbench provide mechanisms for capturing modeling protocols. The Workbench also provides access to the important descriptors as allowed by the various modeling algorithms. In addition, published models include auto generated help text that embeds the model building and validation details. Thus those wishing to utilize the models can understand the finer details of the process. The published models are available by default as calculable properties in Pipeline Pilot and integration with other workflow tools or interfaces is possible through the use of web-services and two additional protocols have been written to this end.

However, the QSAR Workbench is not a black-box system and provides the flexibility to drill down to the detail of any particular step or, indeed, to build individual models in a bespoke manner. Important aspects of the data preparation such as chemistry normalization are handled in a consistent and flexible manner and the use of standard workflows makes the system extensible and highly customizable. Thus, the system enables the model builder to focus on the important aspects of model validation and analysis rather than data manipulation and the requirements of disparate software packages.

As the example applications above have shown the user can then start to answer questions such as: why did this model outperform others? Are there combinations of models and descriptors that are working better? How stable are the models to the pretreatment and parameterization? The results show that with reasonable default settings and protocols the QSAR Workbench can produce models that are equivalent in terms of performance to what might be considered as state-of-the-art models for the specific endpoints considered.

The use of large scale automated modeling also raises several challenges or opportunities. As described earlier, the decision was made at the outset not to include any form of competitive workflow into the model building process. Nevertheless the possibility of chance correlation should not be overlooked. The Workbench enforces good modeling practice and provides a number of features to mitigate this risk, as mentioned in the Implementation section when describing model validation. The emphasis on plots and interactive triage highlights a particular model as being an outlier if other similarly built models all appear worse. The Split algorithms to create Test sets of increasing difficulty also help as it is unlikely that a much better model would be derived from a whole cluster based selection than from say a Randomized or Diverse selection. Indeed the whole cluster (approximating to a leave-class-out validation) has been described as pessimistic by some experts [34]. A completely independent hold out set is used for model validation. The true test of a model is a further ‘completely independent’ and probably temporal set [34] which would be the last gate a model would pass through prior to production deployment. There are additional approaches that can be taken to help identify such chance correlations and the latest version of the Workbench includes options for additional model cross-validation (outside of that used in a particular modeling approach) and Y-scrambling.

The availability of a large number of models from different methodologies provides a pool of models for an ensemble modeling approach. The creation of such models, with increased predictive power, through methods such as data fusion are well established [35]. When models, that have independence in the predicted errors, are combined in this way then the average predictive value should approach the true value as the square of the number of models included in the ensemble. Though such methods offer a clear route to improved predictions, there is almost always an associated reduction in the ability to interpret the resulting ensemble model. This situation is akin to the use of a Random Forest of recursive partitioning trees. The version of QSAR Workbench used in the preparation of this work does not contain any functionality to generate such ensemble models; however we believe that the choice of the Pipeline Pilot framework provides the flexibility to allow this to be rapidly implemented. In fact a recent update to the QSAR Workbench includes an interactive graphical report allowing users to “drill down” to the individual predictions across a sub-set of models, for an external data-set. In this way a manual set of models suitable for combination into an ensemble could be selected, following the general philosophy of the Workbench that the QSAR expert can bring value to the model selection process.

Another important factor when considering the utility of a model in providing predictions to medicinal chemists in an industrial setting is the quantification of the domain of applicability of the model. Ideally a QSAR model prediction would also come with an associated estimate of the error of prediction. In practical usage of global models in a drug discovery program setting, the continued verification of the model performance is good practice. This can highlight systematic variation (a slope or intercept shift) or cases where the global model breaks down and a local model can be built as more information becomes available. Such activities are made more tractable by having an appropriate modeling infrastructure as presented here.

There have been a number of recent efforts to derive quantitative measures of applicability domain of QSAR models [6, 36–38]. Generally these utilize a measure of “distance” to the training set, from simple Euclidean measures to more advanced methods like the Mahalanobis distance. The underlying Pipeline Pilot learner components exploited in the current version of the QSAR Workbench all have the ability to provide some measure of applicability domain along with the prediction. These include warnings when descriptors are out of range of those seen in the training set, or outside the Optimal Prediction Space [39] as well as other measures such as Mahalanobis distance. Provision of estimates of errors in prediction could also be derived from these measures [40]. Currently no explicit reporting of these metrics is performed in the QSAR Workbench. The advantages of an implementation in a workflow tool such as Pipeline Pilot opposed to compiled code in a product are particularly evident in such cases where the science is not well developed and could become fast moving as implementation (and subsequent removal) of one or more such methods is straightforward.

In conclusion, we have presented the QSAR Workbench. A workflow based system for automated high-throughput model building based on local expert specifications. The Workbench interfaces to a range of model building methodologies and provides graphical tools for navigating and triaging the resulting model space. This allows for identification of outlier models or methods that are tending to over-fit in a particular scenario and identification of trends in the data pretreatment (data set splitting) amongst others. The system integrates directly with the chemist desktop through the publication of models as web-services and is extensible and maintainable through the extensive use of Pipeline Pilot workflows to build the main work protocols. The examples presented show that the system is capable of building robust, high quality models in an automated fashion. With this infrastructure in place we are now in a position to exploit the full value from enterprise wide QSAR modeling across all endpoints of interest to drug discovery programs.

## Supporting information

The full list of descriptors and the relevant groupings used in building the models. A full description of the QSAR Workbench system. This material is available free of charge via the link below.

## Electronic supplementary material

Below is the link to the electronic supplementary material.

Supplementary material 1 (DOCX 1046 kb)

## References

[1] Hansch C, Selassie C, John BT, David JT (2007). Comprehensive Medicinal Chemistry II.

[2] Tropsha A, John BT, David JT (2007). Comprehensive Medicinal Chemistry II.

[3] Nicolotti O, Gillet VJ, Fleming PJ, Green DVS (2002). Multiobjective optimization in quantitative structure–activity relationships: deriving accurate and interpretable QSARs. J Med Chem.

[4] Birchall K, Gillet VJ, Harper G, Pickett SD (2008) Evolving interpretable structure-activity relationships. 1. Reduced graph queries. J Chem Inf Model 48:1543–1557 10.1021/ci8000502 18630899

[5] Birchall K, Gillet VJ, Harper G, Pickett SD (2008). Evolving interpretable structure–activity relationship models. 2. Using multiobjective optimization to derive multiple models. J Chem Inf Model.

[6] Tropsha A (2010). Best practices for QSAR model development, validation, and exploitation. Mol Inform.

[7] Cramer RD (2011). Rethinking 3D-QSAR. J Comput Aided Mol Des.

[8] Bruce CL, Melville JL, Pickett SD, Hirst JD (2007). Contemporary QSAR classifiers compared. J Chem Inf Model.

[9] Cartmell J, Enoch S, Krstajic D, Leahy D (2005). Automated QSPR through competitive workflow. J Comput Aided Mol Des.

[10] Rodgers SL, Davis AM, Tomkinson NP, van de Waterbeemd H (2011). Predictivity of simulated ADME AutoQSAR models over time. Mol Inform.

[11] Davis AM, Wood DJ (2013). Quantitative structure–activity relationship models that stand the test of time. Mol Pharm.

[12] Stalring J, Carlsson L, Almeida P, Boyer S (2011). AZOrange—high performance open source machine learning for QSAR modeling in a graphical programming environment. J Cheminform.

[13] Green DVS, Pickett SD, Keefer CE, Bizon C, Woody N, Chakravorty S (2008) Automated predictive modelling: modeller’s utopia or fools’ gold? http://www.soci.org/News/Fine-Chemoinformatics-SAR

[14] Pipeline Pilot (2011) Accelrys Ltd, San Diego. California

[15] Chaudry Q, Piclin N, Cotterill J, Pintore M, Price NR, Chretien JR, Roncaglioni A (2010). Global QSAR models of skin sensitisers for regulatory purposes. Chem Cent J.

[16] Zhao C, Boriani E, Chana A, Roncaglioni A, Benfenati E (2008). A new hybrid QSAR model for predicting bioconcentration factor (BCF). Chemosphere.

[17] Lombardo A, Roncaglioni A, Boriani E, Milan C, Benfenati E (2010). Assessment and validation of the CAESAR predictive model for bioconcentration factor (BCF) in fish. Chem Cent J.

[18] Benfenati E (2010). The CAESAR project for in silico models for the REACH legislation. Chem Cent J.

[19] Helium in Excel: a new paradigm for data insight. http://www.prweb.com/releases/2011/04/prweb5236694.htm Accessed Jan 2013

[20] Shearer C (2002). The CRISP-DM model: the new blueprint for data mining. J Data Warehous.

[21] Harper G, Pickett SD (2006). Methods for mining HTS data. Drug Discovery Today.

[22] http://www.sencha.com/

[23] http://jquery.com

[24] http://www.w3.org/TR/xml/

[25] http://www.ietf.org/rfc/rfc4627.txt

[26] http://www.w3.org/TR/html401

[27] http://en.wikipedia.org/wiki/AJAX

[28] http://www.w3.org/TR/soap/

[29] http://www.w3.org/TR/ws-arch/

[30] Brown CD, Davis HT (2006). Receiver operating characteristics curves and related decision measures: a tutorial. Chemom Intell Lab Syst.

[31] Bi J, Bennett KP (2003) Proceedings of the 20th International Conference on Machine Learning, AAAI Press

[32] http://www.caesar-project.eu/index.php

[33] http://www.oecd.org/document/2/0,3746,en_2649_34379_42926338_1_1_1_1,00.html

[34] Sheridan RP (2013) Time-split cross-validation as a method for estimating the goodness of prospective prediction. J Chem Inf Model. doi:10.1021/ci400084k 10.1021/ci400084k 23521722

[35] Willett P (2006). Enhancing the effectiveness of ligand-based virtual screening using data fusion. QSAR Comb Sci.

[36] Chirico N, Gramatica P (2011). Real external predictivity of QSAR models: how to evaluate it? Comparison of different validation criteria and proposal of using the concordance correlation coefficient. J Chem Inf Model.

[37] Sahigara F, Mansouri K, Ballabio D, Mauri A, Consonni V, Todeschini R (2012). Comparison of different approaches to define the applicability domain of QSAR models. Molecules.

[38] Sheridan RP (2012). Three useful dimensions for domain applicability in QSAR models using random forest. J Chem Inf Model.

[39] Gombar VK (2000) Method and apparatus for validation of model-based predictions. US19960687726 [US6036349 A]. U.S.A

[40] Brown R., Honeycutt D., Aaron S. Quantifying model errors UKQSAR Spring Meeting 2010 http://www.ukqsar.org/slides/Rob_Brown.pdf

[41] Xia X, Maliski EG, Gallant P, Rogers D (2004). Classification of kinase inhibitors using a Bayesian model. J Med Chem.

[42] Breiman L, Friedman J, Stone CJ, Olshen RA (1984). Classification and regression trees.

[43] Venables WN, Ripley BD (2002) Modern applied statistics with S, Springer

[44] Dimitriadou E, Hornik K, Leisch F, Meyer D, Weingassel A (2010) Misc functions of the department of statistics (e1071) http://CRAN.R-project.org/package=e1071

[45] Wehrens R, Mevik BH (2013) Pls: partial least squares regression (PLSR) and principal component regression (PCR) http://mevik.net/work/software/pls.html

[46] Hall LH, Kier LB (1991) The molecular connectivity chi indexes and kappa shape indexes in structure-property modelling. [2], 367–21. New York, VCH Publishers. Reviews in Computational Chemistry. Lipkowitz, K. B. and Boyd, D. B

[47] Rogers D, Hahn M (2010). Extended-connectivity fingerprints. J Chem Inf Model.

[48] Hall LH, Kier LB (1995). Electrotopological state indices for atom types: a novel combination of electronic, topological and valence state information. J Chem Inf Comput Sci.

[49] Hall LH, Mohney B, Kier LB (1991). The electrotopological state: structure information at the atomic level for molecular graphs. J Chem Inf Comput Sci.

[50] Hall LH, Kier LB (2000). The e-state as the basis for molecular structure space definition and structure similarity. J Chem Inf Comput Sci.

