# Supplementary material for: QSAR workbench: automating QSAR modeling to drive compound design
Source: J Comput Aided Mol Des. 2013 Apr 25;27(4):321–36. doi: 10.1007/s10822-013-9648-4 (PMC3657086; doi:10.1007/s10822-013-9648-4)
Supplement: Supplementary file 1 — Supplementary material 1 (DOCX 1046 kb) [file 10822_2013_9648_MOESM1_ESM.docx]

Supporting Information for

QSAR Workbench: Automating QSAR modeling to drive compound design

Richard Cox^1^, Darren V. S. Green^2^, Christopher N. Luscombe^2^, Noj Malcolm^1^*, Stephen D. Pickett^2^*.

GlaxoSmithKline, Gunnels Wood Road, Stevenage, SG1 2NY.

1) Accelrys Ltd., 334 Cambridge Science Park, Cambridge, CB4 0WN, UK

2) GlaxoSmithKline

Corresponding Authors

Noj.Malcolm@accelrys.com, Stephen.D.Pickett@gsk.com

S1. QSAR Workbench Implementation Details

QSAR WORKBENCH IMPLEMENTATION

Application Design Overview

QSAR Workbench is a lightweight Pipeline Pilot web application that provides an intuitive, user centric, sandbox environment for building, validating and publishing QSAR models. Although aimed at workgroup sized teams of users, the application also provides enterprise scale capabilities such as integration points via Web Services for existing corporate modeling applications and workflow capture and replay.

In recent years JavaScript frameworks have emerged as viable technologies for building Rich Internet Applications (RIAs) [1][2]. An RIA is a web application that provides user interface capabilities normally only found in desktop applications. Until recently such applications would typically have been implemented using a browser extension technology such as Flash or Java potentially leading to the code maintenance barriers. QSAR Workbench is an example of a JavaScript based RIA where the majority of the application’s code resides in the client tier whilst the server side layer of the application is simply responsible for providing data (usually formatted as XML [3], JSON [4] or HTML [5]) to the client layer. This application design is commonly referred to as AJAX [6]. QSAR Workbench also makes extensive use of the Pipeline Pilot Enterprise Server as an application server; for example to provide JSON formatted data to the client application, as a scientific modeling platform; to provide services to build and validate models using several learner algorithms and also as a reporting server to return HTML formatted data to the client. The implementation uses the Pipeline Pilot Client SDK (Software Development Kit) which allows communication between the client and Pipeline Pilot via SOAP [7] Web Services [8] and also several extensions to the SDK to provide tight integration with a third party JavaScript library. The workbench also utilizes a custom extension to the Pipeline Pilot reporting collection which allows for flexible client side validation of HTML forms.

QSAR Workbench provides two main views, the first (Figure 1) allows users to manage projects and data sources and provides occasional or non-expert users extremely simple (a few clicks) model building functionality. The second view (Figure 2) provides a more guided workflow where individual tasks commonly performed in QSAR model building processes (such as splitting a data set into training and test sets) are logically grouped together and accessed via a traditional left to right horizontal navigation toolbar. The splitting of the application across two views is simply a design choice, in practice the application resides within a single html page and a ‘link-in’ capability is provided so that individual projects can be bookmarked using e.g. /qswb?name=my_project, visiting such a url then takes the user directly to the second more complex view.


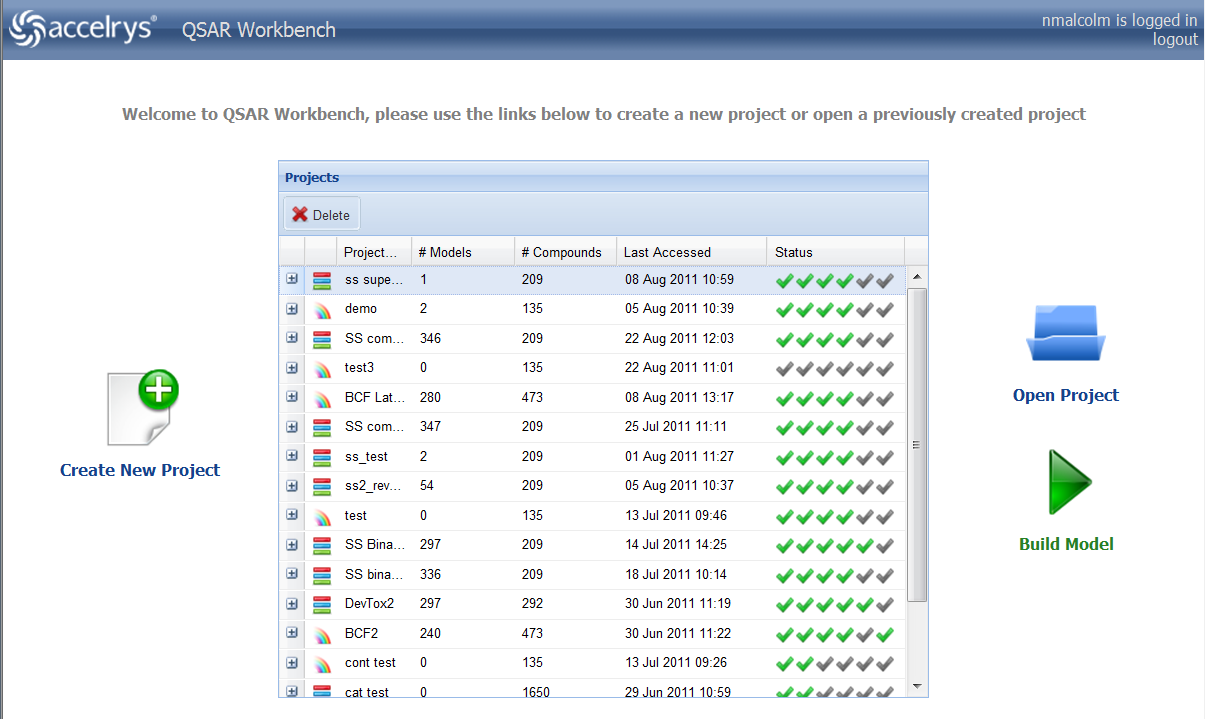


Figure 1. Screenshot showing the QSAR Workbench modeling project management view.

When designing an Ajax JavaScript client one of the most important decisions is what generic behaviors will be built into the application. Figure 2 shows an example of the second view within QSAR Workbench and is used to illustrate some of the behaviors described below. In the design of these features, where possible within the constraints of the project we have attempted to make each feature configurable by editing Pipeline Pilot protocols and/or components so that no expert JavaScript knowledge is required to maintain and extend the application.


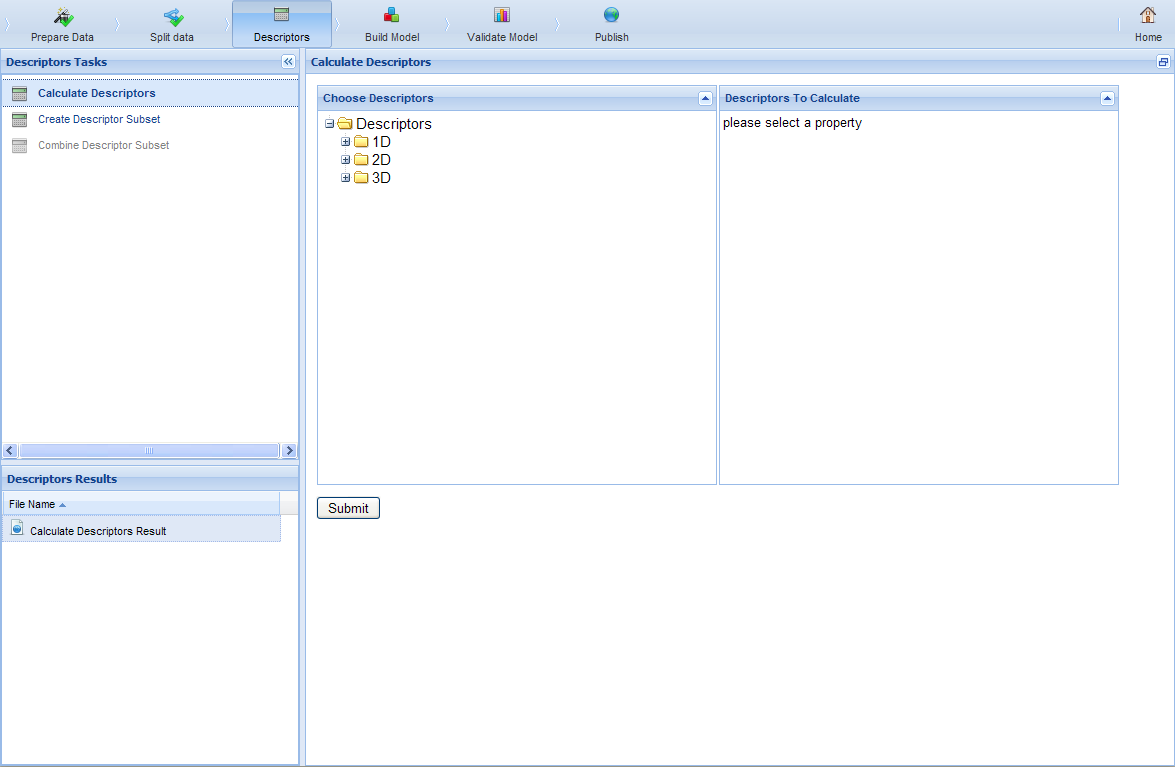


Figure 2 Screenshot showing the QSAR Workbench modeling workflow expert view. This particular section allows for descriptor calculation.

Data Storage

QSAR workbench uses server-side file based storage to store project related data files, HTML reports, model building parameters and other data for each action.

Application workflow

The horizontal ribbon toolbar shown in Figure 2 provides basic navigation behavior whereby clicking one of the task-group buttons switches the view beneath the ribbon to provide the appropriate links to tasks and/or previously viewed reports.

The upper left hand side menu provides links appropriate for the currently selected task group. Clicking one of these links either generates an HTML form configured by the Pipeline Pilot developer or, if a pre-configured form is not provided, a form is automatically generated. Some tasks do not require the user to fill in a form and so are run directly by configuring the task itself as the ‘form’. The links are ordered top to bottom in the order decided most logical during development, the vertical order is controlled by a numbering convention and new links can be added to the application simply by saving a new protocol within the folder for that task group.

A key feature of the guided workflow view is the task dependencies, whereby certain task links are disabled until a previously required task has been completed, for instance you cannot publish a model until you have built a model. To configure dependencies the Protocol developer has to add a parameter with certain legal or allowed values to the task protocol, secondly a component is provided by the framework which must be included in any task protocol that needs to update the dependencies. Every time a task is run in the workbench a JSON string representing the current dependency state is sent to the JavaScript client that automatically enables or disables the task links appropriately.

For each task group one of the tasks can optionally be configured (within a protocol) to be the default task for that group, if there is a default task then that task will be run whenever the task group is activated, this saves the user several clicks and helps to keep attention focused on the central panel.

The bottom left hand panel provides a table view of any files that have been generated by running a particular task. Whenever a task group becomes active the most recently created file is displayed in the central panel. If a file is of a type that cannot be displayed within a webpage (such as a .zip file) a download link is automatically provided instead. Task protocols write any files the developer wishes to be displayed in this view to an agreed location on the Pipeline Pilot server (referred to as the project data directory). In this way files that are generated by running a particular task are persistent and the user can leave and re-visit the project at any stage in the model building workflow.

Pipeline Pilot protocols

The QSAR Workbench relies heavily on a back-end computational framework developed with Accelrys’ Pipeline Pilot [9]. The use of Pipeline Pilot means that the framework is easily modified and extended. The following sections detail the current suite of protocols. Most computational tasks have an associated web form allowing users to modify details of the input parameters.

A key part of the QSAR Workbench framework is the automated auditing of users input to each task, this is achieved through recording of the parameter choices made through the associated web forms. Currently this information is used solely to allow publication of workflows consisting of a chain of tasks, these workflows then become available for replay from the project entry page, through use of the “Big Green Button” - see Figure 1, labeled “Build Model”. It is easy to imagine how this audit information could be further exploited, for example enabling automated generation of regulatory submissions (e.g. QMRF [10] documents for REACH [11]).

Individual tasks are grouped into six areas, details of the tasks within each of these groups as well as the precursor Project Creation are given in the following sections.

Project Creation

A project, as defined within the QSAR Workbench framework, requires three key pieces of information: a data source file containing the chemistry and response data; the name of the end point response data field in the data source file; how the end point data is to be modeled – either as categorical or continuous data. The project creation information is input through a two-step web form (see Figure 3), the first step, allows users to define a unique name for the project, the data source file and the format of the data source file. Currently SD, Mol2, smiles and excel formats are supported, the latter requiring a column containing the chemistry information in smiles format. The second step includes an analysis of the data source file, allowing users to selected the response property field from a drop down list, in addition a preview of the first ten entries in the data source file can be shown. The second step also requires users to select the type of model to build.

Figure 3. Input forms for the project creation task. The top image shows the first form defining the data source file and format. The bottom image shows the form for definition of the response property field and model type selection.

Data Preparation

There are currently three tasks available in the Prepare Data group: Prepare Chemistry; Prepare Response; Review Chemistry Normalization.

Prepare Chemistry allows users to perform common chemistry standardization on the input structures. Currently there are five possible options: Generate 3D Coordinates; Add Hydrogens; Strip Salts; Standardize Molecule; Ionize at pH. The Standardize Chemistry option performs standardization of charges and stereochemistry. The Prepare Chemistry task must be performed before any other tasks in the QSAR Workbench become accessible. Chemistry normalization steps are always performed against the original data source chemistry. Additionally users may choose to visually analyze the chemistry normalization results.

Prepare Response allows users to perform a number of modifications of the underlying response data. For regression type floating point data users may scale the data in five possible ways: Mean Centre and Scale to Unit Variance; Scale To Unit Range; Scale Between One and Minus One; Natural Log; Divide by Mean. For classification models there are three further transforms: Convert to Categorical Response, this allows users to define one or more boundaries to define two or more classes from data that was originally continuous; Create Binary Categories, this allows users to convert multiple category data into just two classes; Convert Integer Response to String. Response Normalization steps are always applied to the original raw response data.

Review Chemistry Normalization allows users to visualize the modifications made to the chemistry with the Prepare Chemistry task. This task may optionally be automatically executed as part of the Prepare Chemistry task.

Data Set Splitting

It is common practice within QSAR to split the data into test and training sets. The training set is passed to the model builder (which may itself use some form of cross-validation in model building) and the test set is used in subsequent model validation. There are currently four individual tasks implemented within the Split Data group: Split Data; Visually Select Split; Analyze Split; Download Splits.

Split Data allows users to split the input data into training and tests sets according to one of six fixed algorithms: Random - the full ligand set is sorted by a random index, the first N compounds are then assigned to the training set according to a user given percentage; Diverse Molecules - a diverse subset of compounds is assigned to the training set according to a user given percentage, diversity is measured according to a user defined set of molecular properties; Random Per Cluster - compounds are first clustered using a user defined set of clustering options, then a random percentage within each cluster is assigned to the training set according to a user given percentage; Individual Clusters (Optimized) - compounds are first clustered using a user defined set of clustering options, then entire clusters are assigned to the training set using an optimization algorithm designed to achieve a user given percentage; Individual Clusters (Random) - compounds are first clustered using a user defined set of clustering options, then a user defined percentage of entire clusters are randomly assigned to the training set; User Defined - compounds are assigned to the training set according to a comma separated list of compound indices, the index correspond to the order the compounds were found in the original chemistry data source. Additionally users may choose to visually analyze the results of the specific split.

Visually Select Split allows users to manually select training set compounds through use of an interactive form, see Figure 4. The form includes visual representation of clustering and PCA analysis of the normalized chemistry space, using both the ECFP_6 fingerprint, and a set of simple molecular properties and molecular property counts.


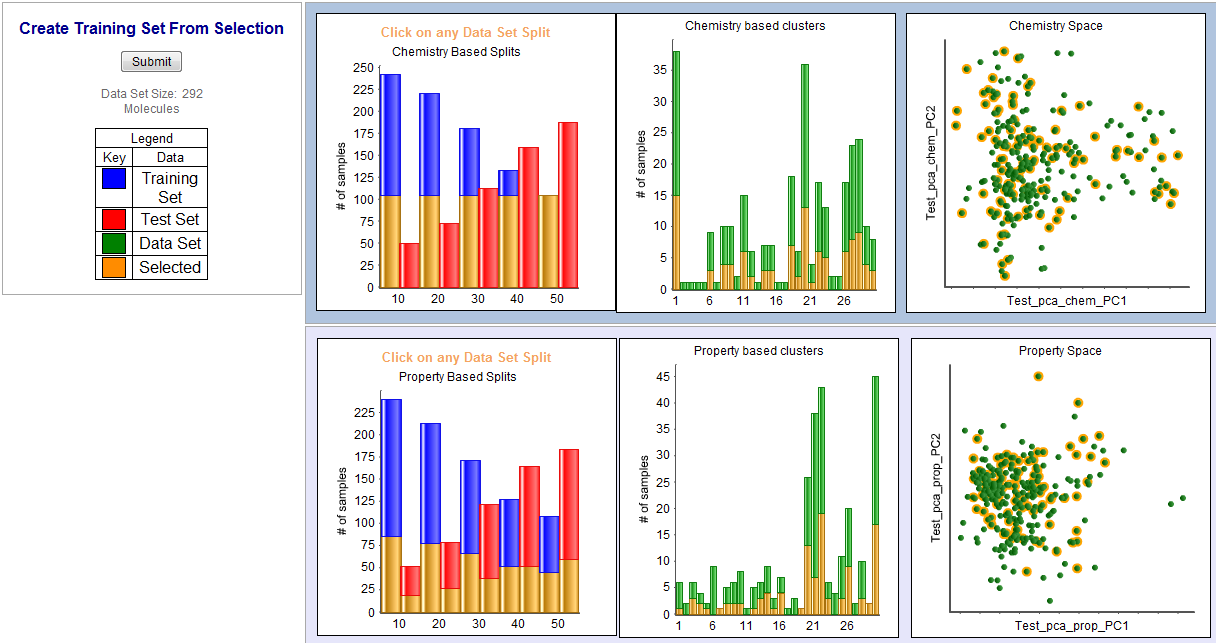


Figure 4. The interactive form used for manual selection of the training and test set.

Analyze Split allows users to visually inspect the effect of one of the currently defined training/test set splits via multi-dimensional scaling plots, see Figure 5. The plots show the differential distribution of training and test set compounds based on distance metrics derived from each of four molecular fingerprints, FCFP_6, ECFP_6, FPFP_6 and EPFP_6. This task may optionally be automatically executed as part of the Split Data task.


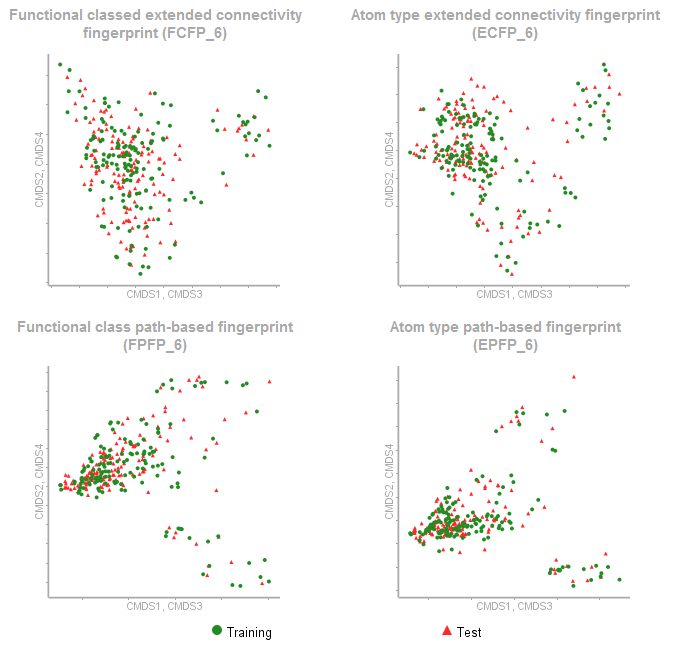


Figure 5. Visual analysis of the training / test set split using the Analyze Split Task.

Download Splits allows users to export details of one of the currently defined training/test set splits to a file on their local computer. The file is in comma separated variable format, users have the option to include any currently calculated descriptors, and whether to export the training set, test set or both. A field is included in the output file indicating which set a particular compound belongs to.

Descriptor Calculation

There are currently three tasks available in the Descriptors group: Calculate Descriptors; Create Descriptor Subset, Combine Descriptor Subset.

Calculate Descriptors allows users to select molecular descriptors to be calculated. The QSAR Workbench stores one global pool of descriptors per project, this task can be used to create the pool of descriptors or add new descriptors to an existing pool. The current version of the QSAR Workbench exposes a relevant subset of the molecular descriptors available in Pipeline Pilot as “calculable” properties. A simple extension mechanism component is also provided that allows Pipeline Pilot developers to extend the descriptors available with custom property calculators such as an alternative logp calculation.

Create Descriptor Subset allows users to manually select a subset of descriptor names from the global pool of calculated descriptors. Any number of subsets can be created and it is these subsets which are used in QSAR model building tasks.

Combine Descriptor Subset allows users to merge one or more existing descriptor subsets into a larger subset.

Model Building

There are currently three tasks available in the Build Model group: Set Learner Defaults; Build Models; Browse Model Reports.

Set Learner Defaults allows users to create parameters sets that modify the fine detail of the underlying Pipeline Pilot learner components. Multiple parameter sets can be defined and their effects explored in combinatorial fashion with the Build Models task.

Build Models allows users to build QSAR models, the task allows building of a single model or automated creation of larger model spaces through combinatorial expansion in four dimensions: training/test set splits; descriptor subsets; learner methods; learner parameters as illustrated in Figure 6. The available model building methods are a subset of those available through Pipeline Pilot, including Pipeline Pilot implementations and methods from the R project [12].


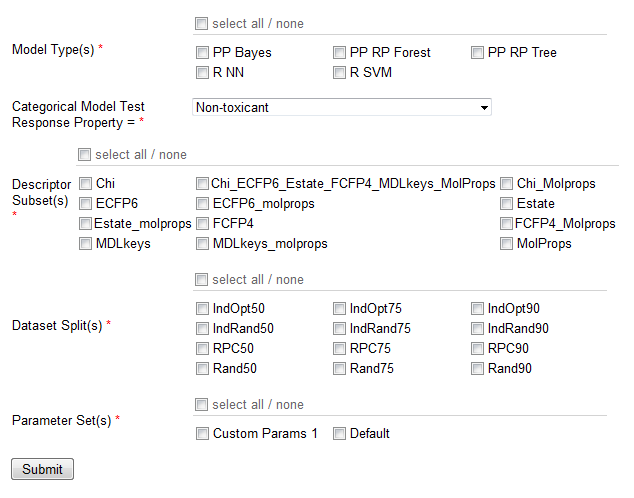


Figure 6. Input form used to define the model building space to be explored by the Build Models task.

For categorical model building users must also select a class from the available list which is considered the “positive” class for the purpose of ROC plot [13] creation and other statistical calculations. On completion of the model building process the Browse Model Reports task is automatically executed and automatically directs the user to the report for the first model built in this step. In addition a summary report of the build process is created containing audit information such as the number of models built, the parameter combination used and the time each model took to build, any model building errors are also captured at this stage.

Browse Model Reports allows users to create a report to browse through detailed results for all currently built models (see Figure 7). For categorical models the report shows ROC plots [13], tabulated model quality statistics and confusion matrices for the training and test sets. For continuous models the report shows Actual vs. Predicted regressions plots, tabulated model quality statistics and REC plots [14] for the training and test sets. For both types of models the report also shows details of model building as well as details of the resulting model. The footer of the report allows rapid paging through results for each currently built model, in addition a PDF report for the current model can be generated.


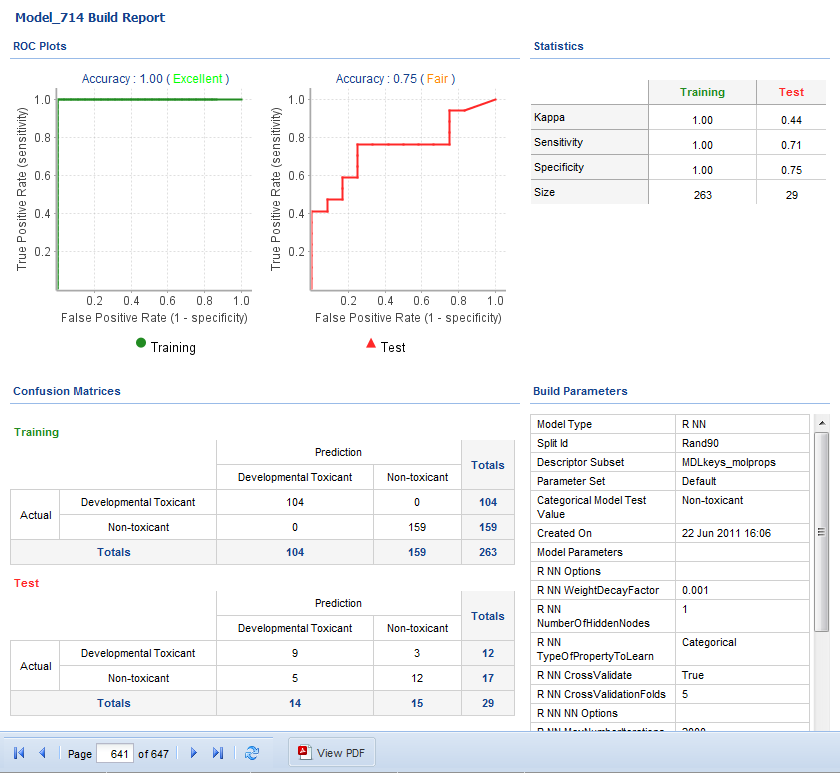


Figure 7. Example model build report, for a categorical end point. Top left, training and test set ROC plots, top right training and test set statistics, bottom left training and test set confusion matrices, bottom right full model building parameter details. The footer provides controls for navigation through current model build reports and export of individual model reports as PDF.

Model Validation

Model validation is a critical aspect of QSAR workflow. There are currently three tasks available in the Validate Model group: Analyze Models; Apply Model to External Set; Browse Prediction Files.

Analyze Models generates an interactive model triage report dashboard. For simplicity, the model triage dashboard has been designed as a two-step process (see Figure 8 and Figure 9) giving users the opportunity to quickly make an initial selection of models by visualizing the current model space for the project and then to further refine the selection in a second drill down view. The model reports include standard plots such as the ROC plot [13] or REC plot [14] as appropriate. For the first step of the model triage dashboard users are presented with an interactive report showing a summary overview of all currently created models. From this report users can easily identify trends, biases, outliers etc. in model space. The interactive nature of the report allows users to select a subset of models for more detailed analysis. The detailed analysis forms step two of the triage, where individual model reports can be compared. From the second step users can “save” one or more models as “favorites” within the QSAR Workbench framework. Additionally a PDF report summarizing the currently selected models can be generated.

Apply Model to External Set allows users to apply a single model to an external validation data set. The model to be applied can be selected either from all currently built models, or if “favorite” models have been saved through the Analyze Models triage process, then just from this subset. The application of the model will automatically apply all appropriate steps used in creation of the selected model, chemistry normalization, descriptor calculation etc. Results can be generated in SD or csv formats and users are given the opportunity to choose which fields will be included in the output file.

Browse Prediction Files allows users to revisit and download results of previous application of the Apply Model to External Data Set task.


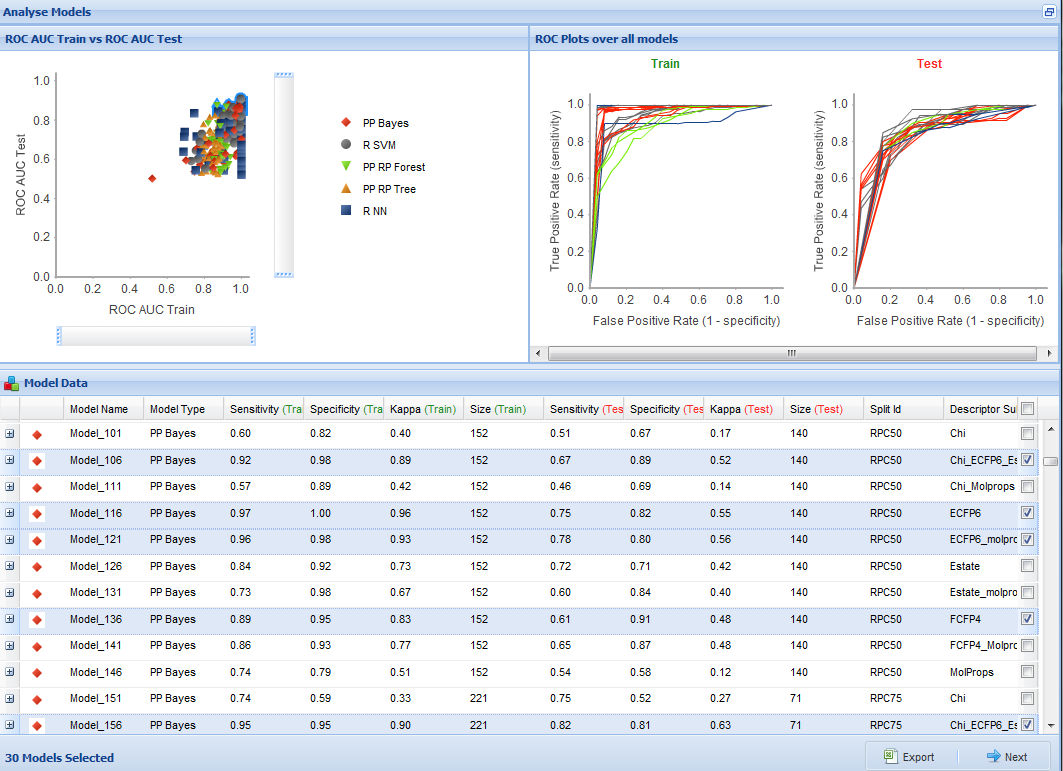


Figure 8. Stage one of the model triage report. The top half of the report shows summary charts over all currently built models. For categorical models the left hand pane shows training set ROC AUC vs. test set ROC AUC, the right hand pane training and test set ROC plots. For continuous models the left hand pane shows training set R^2^ vs. test set R^2^ regression plots and the right hand pane shows REC plots. The bottom half of the report shows a grid over all models. The grid shows summary information on the model building process as well as statistical information on the model. The XY chart in the top left-hand pane, allows users to select models of interest, on selection the chart(s) in the top right hand pane are filtered to only show results for the selected models, in addition models are highlighted and selected in the grid in the bottom panel. The footer of the report allows users to export a csv file with details on currently selected models, and to pass selected models onto the second stage of the model triage process.


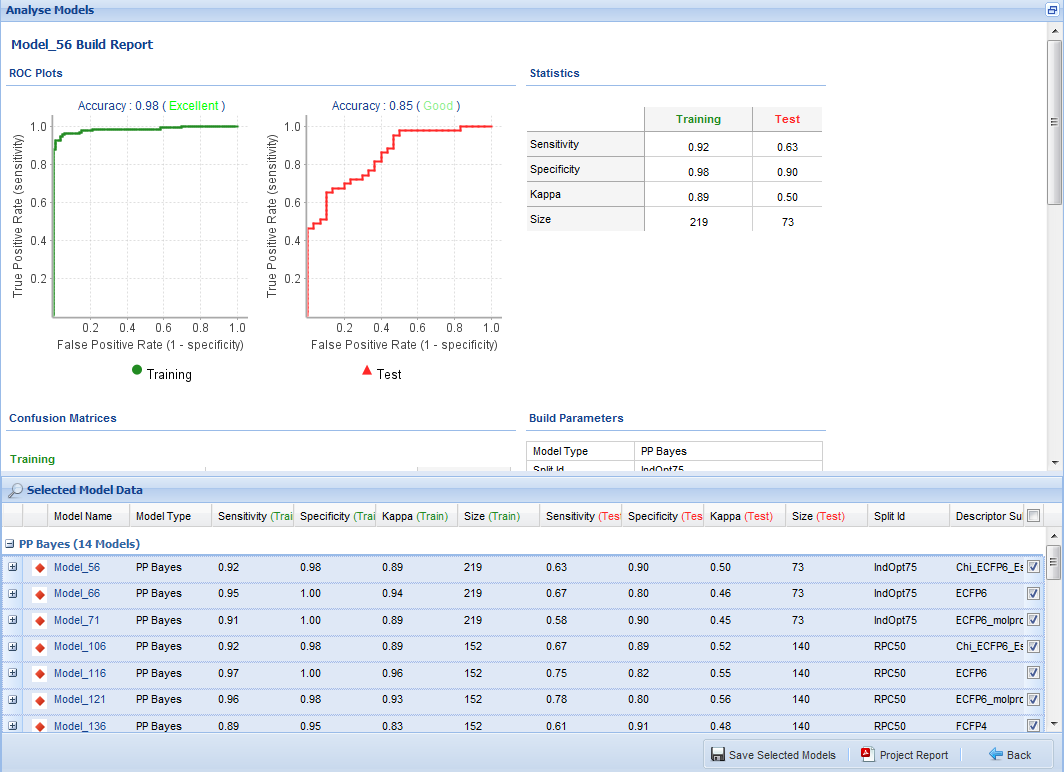


Figure 9. Stage two of the model triage report. The top half of the report shows details of the currently selected model, the individual elements of this section are identical to those available in the Browse Model Report shown in Figure 8. The bottom half of the report shows a grid over all models selected in stage one of the model triage process, clicking on a model name updates the top half of the report to show details for that model. The grid shows summary information on the model building process as well as statistical information on the model. The footer of the report allows users to save a subset of selected models within the framework as “favorites”, generate a PDF report of selected models, or return to stage one of the model triage process.

Model Publication

There are currently three tasks available in the Publish group: Publish Single Model; Publish Method; Browse Published Models.

Publish Single Model allows users to select a single model to be made publically available on the Pipeline Pilot server for production usage. Although published models are truly public by default, administrators can configure access control on a per folder basis using the Pipeline Pilot Administration Portal. Users can give a short name to the published model, which would be used as the name of the “calculable” property on the Pipeline Pilot server. In addition a longer description of the model can be given. An important aspect of model publication that builds upon the implementation is that the help text for the published model component contains the information within the original model reports. Thus the model help text is auto generated. The QSAR Workbench framework also provides two web-services for exploitation of published models, which make for simple integration with existing corporate IT infrastructures. The first web-service returns a list of currently published models, the second will return predictions for a specific published model given a set of smiles strings as input.

Publish Method allows users to make use of the automated auditing provided by the QSAR Workbench framework. While users are exploring model space with the web interface, each step that creates or modifies data is captured in a machine readable format, in the form of a Pipeline Pilot protocol xml. Publish Method exploits these stored protocols allowing the user to define a workflow through selection of one or more previously applied QSAR Workbench tasks. Once captured the workflow steps can be “re-played” against a new project. These workflows can be published privately, only for use by the current QSAR Workbench user, or publically, and thus available to any user on the QSAR Workbench server.

Browse Published Models allows users to see details of the models published from the current project in a tabulated report.

S2. Descriptor Subsets

The following descriptor subsets are available. Individual elements from the list can be selected. The descriptors are all standard Pipeline Pilot properties. The names are those used to refer to the properties in Pipeline Pilot.

Subset Chi

CHI_0

CHI_1

CHI_2

CHI_3_C

CHI_3_CH

CHI_3_P

CHI_V_0

CHI_V_1

CHI_V_2

CHI_V_3_C

CHI_V_3_CH

CHI_V_3_P

Subset ECFP6

ECFP_6

Subset Estate

ES_Count_aaaC

ES_Count_aaCH

ES_Count_aaN

ES_Count_aaNH

ES_Count_aaO

ES_Count_aaS

ES_Count_aasC

ES_Count_aaSe

ES_Count_aasN

ES_Count_dCH2

ES_Count_ddC

ES_Count_ddsN

ES_Count_ddssS

ES_Count_ddssSe

ES_Count_dNH

ES_Count_dO

ES_Count_dS

ES_Count_dsCH

ES_Count_dSe

ES_Count_dsN

ES_Count_dssC

ES_Count_dssS

ES_Count_dssSe

ES_Count_dsssP

ES_Count_sAsH2

ES_Count_sBr

ES_Count_sCH3

ES_Count_sCl

ES_Count_sF

ES_Count_sGeH3

ES_Count_sI

ES_Count_sLi

ES_Count_sNH2

ES_Count_sNH3

ES_Count_sOH

ES_Count_sPbH3

ES_Count_sPH2

ES_Count_ssAsH

ES_Count_ssBe

ES_Count_ssBH

ES_Count_ssCH2

ES_Count_sSeH

ES_Count_ssGeH2

ES_Count_sSH

ES_Count_sSiH3

ES_Count_ssNH

ES_Count_ssNH2

ES_Count_sSnH3

ES_Count_ssO

ES_Count_ssPbH2

ES_Count_ssPH

ES_Count_ssS

ES_Count_sssAs

ES_Count_sssB

ES_Count_sssCH

ES_Count_sssdAs

ES_Count_ssSe

ES_Count_sssGeH

ES_Count_ssSiH2

ES_Count_sssN

ES_Count_sssNH

ES_Count_ssSnH2

ES_Count_sssP

ES_Count_sssPbH

ES_Count_ssssB

ES_Count_ssssBe

ES_Count_ssssC

ES_Count_ssssGe

ES_Count_sssSiH

ES_Count_ssssN

ES_Count_sssSnH

ES_Count_ssssPb

ES_Count_sssssAs

ES_Count_ssssSi

ES_Count_ssssSn

ES_Count_sssssP

ES_Count_tCH

ES_Count_tN

ES_Count_tsC

ES_Sum_aaaC

ES_Sum_aaCH

ES_Sum_aaN

ES_Sum_aaNH

ES_Sum_aaO

ES_Sum_aaS

ES_Sum_aasC

ES_Sum_aaSe

ES_Sum_aasN

ES_Sum_dCH2

ES_Sum_ddC

ES_Sum_ddsN

ES_Sum_ddssS

ES_Sum_ddssSe

ES_Sum_dNH

ES_Sum_dO

ES_Sum_dS

ES_Sum_dsCH

ES_Sum_dSe

ES_Sum_dsN

ES_Sum_dssC

ES_Sum_dssS

ES_Sum_dssSe

ES_Sum_dsssP

ES_Sum_sAsH2

ES_Sum_sBr

ES_Sum_sCH3

ES_Sum_sCl

ES_Sum_sF

ES_Sum_sGeH3

ES_Sum_sI

ES_Sum_sLi

ES_Sum_sNH2

ES_Sum_sNH3

ES_Sum_sOH

ES_Sum_sPbH3

ES_Sum_sPH2

ES_Sum_ssAsH

ES_Sum_ssBe

ES_Sum_ssBH

ES_Sum_ssCH2

ES_Sum_sSeH

ES_Sum_ssGeH2

ES_Sum_sSH

ES_Sum_sSiH3

ES_Sum_ssNH

ES_Sum_ssNH2

ES_Sum_sSnH3

ES_Sum_ssO

ES_Sum_ssPbH2

ES_Sum_ssPH

ES_Sum_ssS

ES_Sum_sssAs

ES_Sum_sssB

ES_Sum_sssCH

ES_Sum_sssdAs

ES_Sum_ssSe

ES_Sum_sssGeH

ES_Sum_ssSiH2

ES_Sum_sssN

ES_Sum_sssNH

ES_Sum_ssSnH2

ES_Sum_sssP

ES_Sum_sssPbH

ES_Sum_ssssB

ES_Sum_ssssBe

ES_Sum_ssssC

ES_Sum_ssssGe

ES_Sum_sssSiH

ES_Sum_ssssN

ES_Sum_sssSnH

ES_Sum_ssssPb

ES_Sum_sssssAs

ES_Sum_ssssSi

ES_Sum_ssssSn

ES_Sum_sssssP

ES_Sum_tCH

ES_Sum_tN

ES_Sum_tsC

Estate_Counts

Estate_Keys

Estate_NumUnknown

Subset FCFP4

FCFP_4

Subset MolProps

ALogP

Molecular_Weight

HBA_Count

HBD_Count

Num_AromaticBonds

Num_AromaticRings

Num_Atoms

Num_Bonds

Num_BridgeBonds

Num_BridgeHeadAtoms

Num_H_Acceptors

Num_H_Acceptors_Lipinski

Num_H_Donors

Num_H_Donors_Lipinski

Num_Rings

Num_RotatableBonds

Num_SpiroAtoms

Num_StereoAtoms

Num_StereoBonds

References

1. http://www.sencha.com

2. http://jquery.com

3. http://www.w3.org/TR/xml

4. http://www.ietf.org/rfc/rfc4627.txt

5. http://www.w3.org/TR/html401

6. http://en.wikipedia.org/wiki/AJAX

7. http://www.w3.org/TR/soap/

8. http://www.w3.org/TR/soap/

9. Pipeline Pilot. 2011. San Diego, California, Accelrys Ltd.

10. http://qsardb.jrc.it/qmrf/help.html

11. http://ec.europa.eu/environment/chemicals/reach/reach_intro.htm

12. R Development Core Team R: A Language and Environment for Statistical Computing*;* R Foundation for Statistical Computing: 2010.

13. Bradley AP (1997) The use of the area under the ROC curve in the evaluation of machine learning algorithms.*Pattern Recognition* 30:1145-1159.

14. Bi J, Bennett KP (2003) AAAI Press,
